# Psychiatric Care Systems Are Not Prepared for the Climate Crisis: A Call for Coordination and Evidence

**DOI:** 10.1177/07067437261428945

**Published:** 2026-03-05

**Authors:** Sean A. Kidd, Siqi Xue, Kwame McKenzie

**Affiliations:** 1 Centre for Addiction and Mental Health, 7938 University of Toronto Department of Psychiatry, Toronto, Ontario, Canada

**Keywords:** climate change, psychiatry, health systems

During the 2021 Western Heat Dome event, the risk of mortality for individuals with schizophrenia was far higher than for any other health condition, amounting to 134 deaths in the hottest 8-day period in British Columbia, Canada.^1^ The majority of those who died had seen a physician for mental health care in the preceding year. Wildfires in 2023 in Alberta and Nova Scotia were associated with a greater prevalence of major depressive disorder, particularly among those with a history of depression.^2^ Such impacts are consistent across jurisdictions and environmental risk conditions and evidenced through a range of methodological approaches.

In this commentary, we argue that psychiatric systems of care in Canada are not sufficiently resilient in the face of our worsening climate, that psychiatry-specific guidance and coordination are both necessary and underdeveloped to date, and we outline promising directions for building climate resilience. Here we apply the definition of a climate resilient health system proposed by the World Health Organization (WHO) as “… those that are capable of anticipating, responding to, coping with, recovering from, and adapting to climate-related shocks and stresses, so as to bring about sustained improvements in population health, despite an unstable climate.”^3^

Psychiatric care system vulnerabilities to climate change are not totally unique and share commonalities with broader medical system challenges (e.g., ambiguity about roles, lack of experience).^4^ There is relevant general health system planning information to be drawn from the United States Climate Resilience Toolkit^5^ and the WHO guidance on resilient health care facilities.^3^ These sources detail approaches to hazard and vulnerability analyses, coordinated warning systems, stress testing, staff training, systems communications, defining and prioritizing interventions, and community partnerships. As resilience planning tools, they will be optimized if applied within needs-based decision-making approaches, with dynamic adaptive models likely being most relevant given the nature of climate change.^6^ Psychiatric care systems have also gained some relevant experience through the COVID-19 pandemic, which required the rapid implementation of modified care protocols, emergency monitoring and response, and coordination at municipal and regional levels. That relevance related to pandemic systems coordination falls short, however, in considering the more periodic nature of climate risks in Canada and their diversity. While there are sources of relevant information, such as those described above, the dots have yet to be connected for psychiatric care systems in Canada.

Health system vulnerabilities to climate change are amplified by several psychiatry-specific circumstances. Examples include: (i) The subtle and overt ways in which psychiatric illnesses affect people's ability to adapt to and recover from weather extremes (e.g., schizophrenia symptoms affecting risk awareness and the ability to take protective actions such as help-seeking).^7^ (ii) A lack of provider awareness about the complexity of environmental risks on mental health that extend far beyond the commonly referenced climate anxiety. Other risk domains include the impacts of weather extremes and air pollution on brain health, cognition, likelihood of engaging in or being exposed to violence, suicidality, and a worsening of existing psychiatric symptoms.^8^ (iii) A compounding of risks due to common physical health comorbidities of psychiatric illnesses, such as cardiac disease and obesity, and the mixed presentations resulting from exposures to weather extremes. Such comorbidities spread responses across both psychiatric and other medical care providers, making system-level coordination and data strategies more complex. (iv) The likely role of stigma related to conditions such as schizophrenia, in which symptoms of heat illness, for example, might be considered psychiatric and not responded to appropriately. Stigma and structural determinants of health corollaries also likely contribute to the limited attention to emergency planning for homeless and other low-income populations, among whom mental illness and addictions are highly prevalent.^9^

Furthermore, when considering how climate change and mental health initiatives are developed and implemented, Canada will have some unique challenges. It will need to work through Federal and Provincial responsibilities and how to build a model which promotes Truth and Reconciliation, that works for urban and rural populations across a vast area and works equitably for a diverse multicultural population. Developing a national plan for psychiatric systems of care, aspects of which are already described in the National Adaptation Strategy, will require several elements: (i) Infrastructure investment to support best practice dissemination, communication and coordination of leadership, and a data strategy—elements relevant to general medical system responses (which are also underdeveloped) alongside psychiatric systems. There may be some promise in considering how Federal health transfers might be tied to, or otherwise encourage, specific outcomes and actions, such as implementing vulnerability assessments and response plans that substantively address psychiatric populations and reducing mortality rates among psychiatric populations during extreme heat events. This is particularly important in a provincial landscape that is very uneven in investment in this area. There are precedents for such federal prioritizing in other areas, such as the national implementation of Integrated Youth Services, where direct funding to initiatives, bilateral agreements, and research funding encourage adoption. (ii) The implementation and testing of responses unique to psychiatric care environments, such as risk assessments accounting for mental illness and addictions, focussed provider and leader education, and intervention strategies tailored to particularly high-risk groups (e.g., socially isolated individuals with Autism and comorbid physical disabilities). Those strategies could include models of outreach during extreme weather events, trauma screening and suicide risk assessment, and psychological first aid. (iii) Lastly, research in psychiatry can be extremely important in generating informed investments in responses. Alongside examining distress related to climate change, we need to increase attention to topics such as the brain development implications of climate change, which have major social and economic impacts. There is also a need for intervention research to balance out an evidence base focussed primarily on descriptions of risk. Most importantly, there is a need for rapid evidence-implementation cycles similar to those seen during the COVID-19 pandemic. Such work will involve improved data infrastructures and implementation scientists collaborating with climate scientists who can ensure that what is being tested is indexed to future pressures through predictive modelling given mounting risks and complexity year over year. As well, the breadth of disciplinary and sector collaborations required is particularly broad in the frame of climate change. Examples include the importance of physiological evidence (e.g., schizophrenia and thermoregulation) in both articulating risk and rigorously testing interventions, and input from engineers and architects on how built environment exposures compound risks (e.g., multitenant housing). Partnerships with lived experience experts, caregivers, and community organizations are also critical to ensure the local relevance of responses and their iterative cycles informed through data sources that often are not accessed through current medical information infrastructures.

Such work in Canada can then connect back to internationally coordinated activities and sources of guidance, such as the WHO, the Belem Health Action Plan, and the Intergovernmental Panel on Climate Change. There is also an imperative, complex as it is in our current political context, for collaboration with the United States, given our shared environmental risk profile. As psychiatric care practitioners and leaders in this area, we see, amidst evidence gaps and many duplicative efforts, an opportunity for Canada to demonstrate leadership—work that can offset the profound and mounting impacts of climate change on the mental health of Canadians.
